# Drowsiness following ondansetron given the day after general anesthesia for postoperative nausea: a pediatric case report

**DOI:** 10.1186/s40981-023-00599-8

**Published:** 2023-02-09

**Authors:** Machiko Furuta, Kiyoyuki Miyasaka, Yasuyuki Suzuki

**Affiliations:** Department of Critical Care and Anesthesia, National Center for Child Health and Development, 2-10-1 Okura, Setagaya-ku, Tokyo, 157-8535 Japan

**Keywords:** Pediatric anesthesia, Ondansetron, Drowsiness, Adverse side effect, PONV

## Abstract

**Background:**

Ondansetron is an antiemetic drug that is useful not only for prevention but also for the treatment of postoperative nausea and vomiting (PONV). We report a rare case of drowsiness in a child after using ondansetron for nausea on the day after general anesthesia.

**Case presentation:**

A 5-year-old boy underwent circumcision under general anesthesia and suffered from postoperative nausea and vomiting. He was administered 0.1mg/kg of ondansetron in the PACU and 23 h later on the day after surgery. After the second dose, he acutely exhibited drowsiness which resolved in 3 h. He was discharged to home later on the same day. He was not given any other drugs at the time, and the drowsiness was thought to be directly attributable to ondansetron, though the exact mechanism was unknown.

**Conclusions:**

When drowsiness or other cognitive symptoms are observed after administration of ondansetron, it must be considered and managed as a possible side effect.

## Background

Postoperative nausea and vomiting (PONV) is a common adverse event after general anesthesia. It occurs in 24–70% [1–3] of pediatric patients. Ondansetron is an antiemetic drug that is considered the “gold standard” for PONV [4]. It is useful not only for prevention but also for the treatment of PONV without causing drowsiness [5]. We report a rare case of drowsiness in a child after using ondansetron for nausea on the day after general anesthesia. Written consent was obtained from the child’s guardian for this case report.

## Case presentation

A boy aged 5 years and 4 months, with a height of 101.2 cm and weight of 15.1 kg, was scheduled for circumcision under general anesthesia to treat true phimosis. He had a history of chronic granulomatosis and had undergone allogenic bone marrow transplantation when 4 years old. Over the course of treatment, he had general anesthesia five times using propofol and remifentanil and experienced no postoperative nausea. He also had no family history of PONV. The preoperative examination was otherwise unremarkable, aside from chronically slightly elevated AST (46 IU/L).

The patient was admitted to the operating room without premedication. Slow induction with sevoflurane and nitrous oxide was performed, and after intravenous access was established, intubation was performed using a supraglottic device. Anesthesia was maintained with oxygen, air, 0.9–1.1% of sevoflurane, and 0.4–0.5 mcg/kg/min of remifentanil during surgery. Sacral epidural anesthesia with 1ml/kg of 0.2% ropivacaine and 15mg/kg of intravenous acetaminophen was administered for postoperative analgesia. The operation time was 1 h and 2 min, and the anesthesia time was 1 h and 43 min.

The patient complained of nausea in the PACU and was administered 0.1mg/kg of ondansetron. After the nausea resolved, he was transferred to the general ward.

The patient was able to drink water at 1 h and 2 h after transfer to the ward, but intravenous infusion was continued because he did not feel well enough to eat dinner. He vomited after breakfast the following day and complained of continued nausea. Discharge to home was postponed, and 0.1mg/kg ondansetron was re-administered—23 h had elapsed since the first dose in the PACU. Thirty minutes after re-administration, he became drowsy and showed only a slight response to stimulation, scoring 5 on the Ramsay sedation scale. Since vital signs were stable with no respiratory depression or need for oxygen, follow-up was continued in the ward with a pulse oximeter.

Three hours after re-administration, the patient recovered to a score of 3 on the Ramsay sedation scale. He could have water without nausea or vomiting. He was discharged later on the same day at the strong request of his guardian. Because the drowsiness improved spontaneously, no additional testing was performed. After discharge from the hospital, he was able to have meals as usual, and drowsiness was not observed.

## Discussion

We experienced drowsiness as a rare adverse effect of ondansetron used in the treatment of PONV on the day after surgery. The patient had two risk factors for PONV (age over 3 years and duration of a surgery over 30 min), which raises PONV risk to about 30% [2]. Trammer et al. verified the efficacy of ondansetron in a systematic review and found that liver enzyme elevation (approximately 3.2%, the number need to harm: NNH31) and headache (approximately 2.7%, NNH36) were the most common adverse events, but did not report drowsiness [6]. With regard to children in this study, one article reported a 35% incidence of headache and one article reported bradycardia in three patients, but no reports of drowsiness.

Patel et al. examined the safety of 5-HT3 inhibitors in combination with general anesthetics in 429 children aged 2–12 years [7]. They reported postoperative drowsiness/sedation in 2% of the ondansetron group, but the frequency was not significantly different from the placebo group. In another placebo-controlled study of 670 children, two children in the ondansetron administration group exhibited postoperative agitation but none had drowsiness [8]. In these studies, it is difficult to distinguish whether drowsiness was a residual effect of general anesthesia or caused by ondansetron itself. In our case, the drowsiness also could have occurred after the first administration, but may not have been recognized post-anesthesia. The drowsiness after the second administration was thought to be directly attributable to ondansetron, because the patient received ondansetron only and no other drugs were administered on that day.

A previous study suggested that patients with severe hepatic impairment should limit their ondansetron dosage because clearance of ondansetron is hepatic [9]. Although AST was slightly elevated in our case, other liver enzyme levels were within normal limits and did not change significantly over time or after the procedure. Therefore, we believe it is unlikely that the observed events were induced by abnormal hepatic metabolism.

Drowsiness persisted for about 3 h, which is consistent with the half-life of 2.6 h for ondansetron administered to children aged 3–7 years [10]. The blood concentration of ondansetron drops below detectable levels 8 h after administration to children under 7 years old [10].

Ondansetron has an antiemetic effect by inhibiting serotonergic activity [11]. Cognitive symptoms may be associated with serotonin toxicity, but this is unlikely to occur acutely following the administration of an antagonist only. Therefore, the exact mechanism of drowsiness caused by ondansetron in this case is unknown. It is possible that he fell asleep as his nausea improved, but the level of sedation observed was beyond physiological sleep, with only a slight response to stimulation.

Although the possibility that drowsiness was induced by an improvement of nausea cannot be completely ruled out, we felt it worthwhile to report that ondansetron may directly or indirectly produce such a response.

## Conclusions

Ondansetron is useful for the prevention and treatment of PONV. However, when drowsiness or other cognitive symptoms are observed after administration, it must be considered and managed as a possible side effect.

## Data Availability

Data sharing is not applicable to this article as no datasets were generated or analyzed during the current study.

## Abbreviation

PONV: Postoperative nausea and vomiting
